# Genomic characterization of *Bacillus cereus* isolated from food poisoning cases revealed the mechanism of toxin production

**DOI:** 10.3389/fmicb.2023.1238799

**Published:** 2024-01-12

**Authors:** Qian Zhou, Guanqiao Li, Yinshan Cui, Jingshu Xiang, Shu Zhu, Shijun Li, Jingyu Huang, Yafang Wang, Ying Liu, Li Zhou

**Affiliations:** ^1^Guizhou Provincial Centre for Disease Control and Prevention, Guiyang, Guizhou, China; ^2^College of Bioinformatics, Chongqing University of Post and Telecommunications, Chongqing, China; ^3^Yunnan Pulis Biotechnology Co., Ltd., Kunming, Yunnan, China

**Keywords:** *Bacillus cereus*, comparative genomic analysis, virulence factor, metabolic pathway, foodborne outbreak

## Abstract

**Introduction:**

*Bacillus cereus* is a ubiquitous opportunistic human pathogen that causes food intoxications worldwide. However, the genomic characteristics and pathogenic mechanisms of *B. cereus* are still unclear.

**Methods:**

Here, we isolated and purified nine strains of *B. cereus* (*LY01-LY09*) that caused vomiting, diarrhea and other symptoms from four foodborne outbreaks happened in Guizhou Province in southwest China from June to September 2021. After colony observation, Gram staining, microscopic examination and biochemical test, they were identified as *B. cereus*. The genomic characteristics, phylogenetic relationships and virulence factors of the isolated strains were analyzed at the genome level. Genome sequencing, comparative genomic analysis, secondary metabolite analysis and quantitative PCR were utilized to give a thorough exploration of the strains.

**Results:**

We obtained the genome maps of *LY01-LY09* and found that *LY01-LY09* had a complex interspecific relationship with B. anthracis and *B. thuringiensis*. We also observed a contraction of gene families in *LY01-LY09*, and the contracted families were mainly associated with prophage, which contributed to the species diversity of *B. cereus*. The *Hsp20* gene family underwent a rapid evolution in *LY01-LY09*, which facilitated the adaptation of the strains to adverse environmental conditions. Moreover, the *LY01-LY09* strains exhibited a higher copy number in the non-ribosomal polypeptide synthetase (NRPS) genes and carried the complete cereulide synthetase (*ces*) gene cluster sequences. Considering that the *NRPS* system is a classical regulatory mechanism for emetic toxin synthesis, we hypothesized that *LY01-LY09* could synthesize emetic toxins through the regulation of *ces* gene clusters by the *NRPS* system.

**Discussion:**

These findings are important for further investigation into the evolutionary relationship between *B. cereus* and their related species, as well as the underlying mechanisms governing the synthesis and secretion of bacterial toxins.

## 1 Introduction

Foodborne pathogens frequently cause public health emergencies (Josephs-Spaulding et al., 2016; Antunes et al., 2020) and have become a critical concern in food safety in China (He and Shi, 2021). Pathogenic microorganisms can lead to both food poisoning and contamination. Microbial contamination poses a significant threat to food safety and the issue of foodborne illnesses resulting from such contamination is also highly critical (Tran et al., 2011; Pires et al., 2021). *B. cereus* is a frequently encountered foodborne pathogen, and *B. cereus*-related food poisoning primarily manifests as acute gastroenteritis characterized by symptoms such as vomiting, fatigue, nausea, and diarrhea (Kotiranta et al., 2000). *B. cereus* causes two distinct types of food poisoning based on symptoms: food intoxication (vomiting type) and food toxico-infection (diarrhea type). According to relevant statistics, the vomiting type accounts for 75.9% of *B. cereus* poisoning cases, while the diarrheal type 11.4%, thus making vomiting the predominant manifestation of *B. cereus* foodborne illness (Zhou et al., 2008). The diarrheal type is mainly attributed to the *B. cereus* production of enterotoxins, including hemolysin BL (HBL), non-hemolytic enterotoxin (NHE), and cytotoxin K (CytK) (Dietrich et al., 2021). In contrast, the vomiting type is caused by cereulide, an emetic toxin produced by *B. cereus* through a non-ribosomal polypeptide synthetase (NRPS), which is encoded by the cereulide synthetase (*ces*) gene cluster (Marxen et al., 2015; Carroll and Wiedmann, 2020). The *ces* gene cluster, about 24 kb long, comprises seven genes, and is typically situated on a megaplasmid of *B. cereus* (Ehling-Schulz et al., 2006). The *B. cereus*-caused vomiting type of food poisoning is often mild, although severe and even lethal conditions might happen in rare cases (Stenfors Arnesen et al., 2008).

Genome sequencing technology allows for a comprehensive analysis of the molecular biological characteristics of pathogenic bacteria at the genetic level, and is widely used in epidemiological investigations of foodborne disease outbreaks (Li et al., 2021). This technology provides a crucial foundation for molecular epidemiological investigations of foodborne disease outbreaks caused by *B. cereus*, enabling not only pathogen identification, but also the detection and classification of virulence and drug resistance genes, and further the analysis of pathogenicity (Balloux et al., 2018). A previous study detected hemolytic (*hblA*, *hblC*, and *hblD*) and non-hemolytic (*nheA*, *nheB*, and *nheC*) enterotoxin genes in two *B. cereus* strains isolated from indoor air, suggesting that airborne isolates may cause diarrhea rather than vomiting (Premkrishnan et al., 2021). Toxigenic heterogeneity was found to be related to toxin genes such as *nheABC*, *hblCDAB*, *cytK2*, *entFM*, and *CesB* in the genomes of *B. cereus sensu lato* (s.l.) isolated from ready-to-eat foods and powdered milk (Sánchez Chica et al., 2020). The characterization of the genomes of *B. cereus* and the identification of virulence-related genes are of great significance for determining the causes of foodborne poisoning. However, few studies have dealt with the genomic profiling of *B. cereus* isolated from food sources that induces symptoms such as vomiting and diarrhea.

In this study, we performed genome sequencing and comparative genetic analysis on nine strains of *B. cereus* (*LY01-LY09*) isolated from contaminated food linked to foodborne outbreaks in Guizhou Province, China to analyze the evolution of these strains at the genome level, explore their virulence factors, and evaluate their pathogenic potential. By analyzing the genomic information of *LY01-LY09*, our objective was to establish a fundamental basis for research on the pathogenic mechanisms of *B. cereus*, as well as for the development of antibiotics, disease prevention and control. This study is of great significance in comprehending the evolutionary traits and patterns of *B. cereus*, and may provide a rapid method for the detection of *B. cereus* strains.

## 2 Materials and methods

### 2.1 Outbreak investigation

From June to September 2021, four foodborne outbreaks had occurred in Ziyun County and Tongren City, Guizhou Province. Persons affected reported symptoms of gastroenteritis. Afterward, technicians from Guizhou Provincial Centre for Disease Control and Prevention carried out epidemiological and environmental investigations. Food samples were collected for laboratory test.

In the 23 cases, more than 86% experienced nausea or vomiting (Supplementary Table 1). Few reported abdominal pain, diarrhea, dizziness, fatigue, etc. In the three outbreaks happened in Ziyun County, all persons had eaten rice noodle (a popular local food) bought from the same vendors or stores. The Tongren outbreak occurred in a nursery, and all affected children had eaten meals served by the nursery kitchen (Supplementary Table 1). By laboratory test, *B. cereus* colonies were isolated from food samples. In addition, two out of four vomit samples from children of the Tongren outbreak yielded toxin-producing *B. cereus*. All those affected were managed symptomatically, except for six were hospitalized and given intravenous rehydration.

### 2.2 Isolation, culture, and identification of bacterial strains

Food samples were approximately diluted, and the diluted solution was inoculated on Mannitol-Egg Yolk-Polymyxin (MYP) medium (Tryptone 10 g/L, Mannitol 10 g/L, Meat extract 1 g/L, NaCl 10 g/L and Phenol red 0.025 g/L) and Nutrient Agar (NA) medium. Number of colonies was enumerated after culturing at 37°C for 24 h (Anon, 1995). Five bacterial colonies from each plate were selected and inoculated on an NA inclined surface for pure culture at 37°C for 24 h. After being confirmed as Gram-positive, the morphology of the bacteria was observed under microscopic examination. Finally, the EasylD Biochemical Identification Kit for *B. cereus* (HuanKai microbial, China) was utilized to identify strains presenting in contaminated food.

### 2.3 Sequencing and assembling of *B. cereus* strain genomes

The genomic DNA of *B. cereus* strains was extracted using SDS, and total DNA was fragmented using the Covaris ultrasonic breaker (Covaris, East Sussex, UK). The library was constructed by the NEBNext^®^Ultra™ DNA Library Prep Kit (NEB, USA) in accordance with the manufacturer’s protocol. After a thorough quality inspection, the library was sequenced by Illumina NovaSeq 6000 platform (Illumina, San Diego, CA, USA) to obtain raw data, which were then filtered to acquire clean data. SOAP *de novo* (v2.04), SPAdes (v 3.15.5), and ABySS (v 2.0) assembly software were utilized for *de novo* assembly of high-quality sequencing data obtained from the *B. cereus* strains, while Contig Integrator for Sequence Assembly (CISA) software (Lin and Liao, 2013) was employed to obtain preliminary assembly results. The assembly output was optimized using GapClose (v1.12), which was employed to fill gaps and improve genome quality.

### 2.4 Genome annotation and collinearity analysis

GeneMarkS (Version 4.17) software was utilized to predict coding genes in the *B. cereus* strains, which were mapped to functional databases (E-value ≤ 1e^–5^) in Kyoto Encyclopedia of Genes and Genomes (KEGG),^1^ Transporter Classification Database (TCDB),^2^ pathogen–host interactions database (PHI),^3^ and carbohydrate active enzyme (CAZy)^4^ to obtain functional annotation of the protein-coding genes. In addition, repeated and tandem repeats were identified using RepeatMasker (Version open-4.0.5) and TRF (Tandem Repeats Finder, Version 4.07b), respectively. To search for homology, BLASTP software (Version 2.12.0+) was utilized to compare the protein-coding genes among the *LY01-LY09* strains. The GENESPACE package of R language was employed for the visualization of genome collinearity.

### 2.5 Gene family clustering and phylogenetic tree construction

**FIGURE 1:**
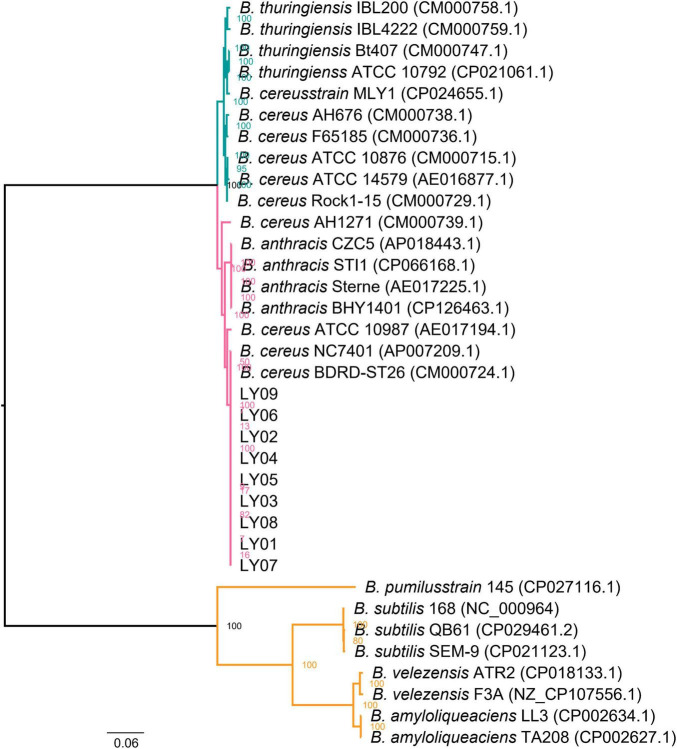
Phylogenetic analysis of the 9 *Bacillus* isolates and 26 *Bacillus* strains downloaded from databases based on whole genome sequence. Strain names and accessions of the downloaded strains are available in the figure. Bootstrap frequencies were obtained with 1,000 replicates.

Furthermore, the complete genome sequences of 26 *Bacillus* strains were downloaded from the NCBI GenBank database, including four *B. anthracis*, two *B. amyloliquefaciens*, four *B. thuringiensis*, one *B. pumilusstrain*, ten *B. cereus*, two *B. velezensis*, and three *B. subtilis*. Strain names and accession numbers of the reference sequences are available in Figure 1. Altogether, 1269 single-copy orthologs were obtained from the whole-genome sequences of the 35 *Bacillus* strains (9 isolated and 26 downloaded) using OrthoFinder (v.2.5.4) (Emms and Kelly, 2019). Multiple alignments of amino acid sequences of orthologs were carried out by MUSCLE (v.3.8.1551) (Edgar, 2004). Conserved blocks from multiple alignments of protein sequences were obtained by Gblocks (Castresana, 2000). Maximum likelihood (ML) tree was constructed using RAxML v.8.2.12 software (Stamatakis, 2014) with 1000 bootstrap replicates by PROTGAMMAILGF model, which was built using ProtTest (v.3.4.2) software (Darriba et al., 2011). Tree visualization was completed by FigTree (v.1.4.4) software. The expansion and contraction analysis of gene family was performed by CAFE (v.4.2.1) software, afterward representative species on the branch were selected.

### 2.6 Virulence factor prediction of the *B. cereus* strains

Protein sequences of the nine *B*. *cereus* strains were subjected to BLAST with an E-value threshold ≤ 1e^–5^ against the virulence factor database (VFDB)^5^ (Liu et al., 2022). The resulting data were then integrated with corresponding functional annotations for each identified virulence factor and its target species, obtaining comprehensive annotation results for the virulence factors of the nine *B*. *cereus* strains. *NRPS* gene sequences were identified by BLAST and motif prediction were performed using the MEME website.^6^ The phylogenetic relationship of *NRPS* gene family was constructed based on IQ-TREE (v. 2.1.4-beta) according ML method.

### 2.7 Verification of virulence factors by q-PCR

The expressions of *hblC, nheB* and *cesB* of the nine *B*. *cereus* strains were detected by quantitative polymerase chain reaction (q-PCR). Specifically, primers and probes were designed based on *B. cereus* virulence factor genes (*hblC, nheB, cesB*). During PCR amplification, TaqMan probes were hydrolyzed by the action of Taq DNA polymerase to generate fluorescence signals. Cycle threshold (Ct) values ≤ 30 were regarded as positive. Amplification curves were obtained for the detection of *B. cereus* virulence factors genes.

### 2.8 Comparative genome analysis

The fastANI program (Jain et al., 2018) was used to calculate the average nucleotide identity (ANI) between the whole genome sequences of two *B. cereus* strains. In addition, MUMmer (v. 4.0.0beta2) (Kurtz et al., 2004) was used to analyze the single nucleotide polymorphism (SNP) and InDels among strains based on the whole genome sequence, with the genome of LY03 as a reference. CIRCOS (v.0.69-9) software (Krzywinski et al., 2009) was used to visualize SNP.

## 3 Results

### 3.1 Identification and characterization of isolates

A total of nine strains of bacteria were isolated and purified from food samples. The selective medium MYP was used to screen and identify the strains. White colonies surrounded by pink areas were observed on the MYP medium, while colonies formed rough white surface with irregular edges on the NA medium (Supplementary Figure 1A). This was consistent with the typical characteristics of *B. cereus*. The nine strains were stained with Gram’s stain and identified as Gram-positive (Supplementary Figure 1B). Finally, the strains were tested for starch hydrolysis, glucose fermentation, Voges-Proskauer (V-P) reaction, etc., using the identification kit. A diffuse growth out into the medium along and away from the stab was observed in the motility test, thus excluding the possibility of *B. mycoides* strains (Supplementary Table 2). Based on the aforementioned testing results, the strains isolated and purified in this study were identified as *B. cereus* according to the National Standard of the People’s Republic of China GB 4789.14 (2014).

### 3.2 Genome structure and linkage map of the *LY01-LY09* strains

The nine strains of *B. cereus* had similar genome sizes, with all but the *LY08* strain (5.68 Mb) possessing a genome size of 5.64 Mb. The GC content ranges from 35.23 to 35.25%, exhibiting a small variation as well. The lengths of their coding genes ranged from 4.75 Mb to 4.79 Mb, accounting for approximately 84.3–84.4% of their respective genome sizes (Supplementary Table 3). The proportions of repeat sequences (LINE, RC, SINE, and LTR) in each genome were similar among the strains (Supplementary Figure 2A). The number of transfer RNAs in each strain ranged from 92 to 93, with a length of 7,000–7,200 bp. Meanwhile, the number of small RNAs varied from 5 to 6, with lengths ranging from 400 to 550 nucleotides (Supplementary Figure 2B). CRISPR (clustered regularly interspaced short palindromic repeat sequences) elements counts ranged from 10 to 29, with lengths varying between 2,476 and 6,175 bp. Furthermore, the nine strains had similar numbers of CRISPR, genomic island (Gis) and prophage (Supplementary Figure 2C). Based on the KEGG database, the metabolic pathways of the *LY01-LY09* cellular gene products and compounds were assigned to six categories: metabolism, genetic information processing, environmental information processing, cellular processes, organismal systems, and human diseases. At level B, a total of 41 metabolic pathways were involved, with a primary focus on amino acid metabolism, carbohydrate metabolism, membrane transport, caffeine and vitamin metabolism, as well as energy metabolism (Supplementary Figure 3A). Functional analysis of the transport proteins in the nine isolates was performed using the TCDB database. We found that the transport proteins were mainly primary active transporters and electrochemical potential-driven transporters (Supplementary Figure 3B). Based on the CAZy database, glycosyltransferase (GTs), Glycoside hydrolases (GHs), carbohydrate esterases (CEs) and auxiliary activities (AAs) were detected as the four enzyme classifications (Supplementary Figure 3C). In addition, we noticed that reduced virulence and unaffected pathogenicity were the phenotypes with the most matched genes (Supplementary Figure 3D).

### 3.3 Comparative genome analysis of the *LY01-LY09* strains

Pairwise macrosynteny analysis revealed a high collinearity among the nine strains, with high conservation at the genome level, except for contig rearrangements on contig3 of *LY02*, and contig3 and contig6 of *LY01* (Supplementary Figure 4). A further analysis detected a fission phenomenon on contig7 and contig9 only, compared with a high collinearity among the isolated strains and the reference *B. cereus* strain. The genomic characteristics of the nine strains of *B. cereus* were compared with each other, and the results showed that pairwise ANI values were greater than 99.98% (Supplementary Table 4). This may indicate that these nine strains were the same strain, which was consistent with the results of collinear analysis. In addition, the genomic sequences of the other eight strains were compared with *LY03* to obtain mutation information (SNPs and InDels). We found few mutation sites between the genomes of the nine strains, indicating their low level of heterogeneity (Supplementary Figure 5).

### 3.4 Phylogenetic analysis based on whole genome sequencing

The evolutionary relationships between the nine isolated strains with other related *Bacillus* strains were analyzed using whole-genome ML phylogenetic tree (Figure 1). The *LY01-LY09* strains showed 100 bootstrap frequency rooted at the branch. The *LY01-LY09* strains exhibited a close genetic relationship with *B. anthracis* and *B. thuringiensis*, which formed sister groups with *B. anthracis* (Figure 1). In addition, *B. thuringiensis*, *B. cereus* and *B. anthracis* were in the same branch, the boundaries between some *B. cereus* and *B. anthracis* strains were not clear. The results showed that the genomes of the three *Bacillus* species were highly similar.

### 3.5 Regulatory networks of the *LY01-LY09* strains and identification of key genes

**FIGURE 2:**
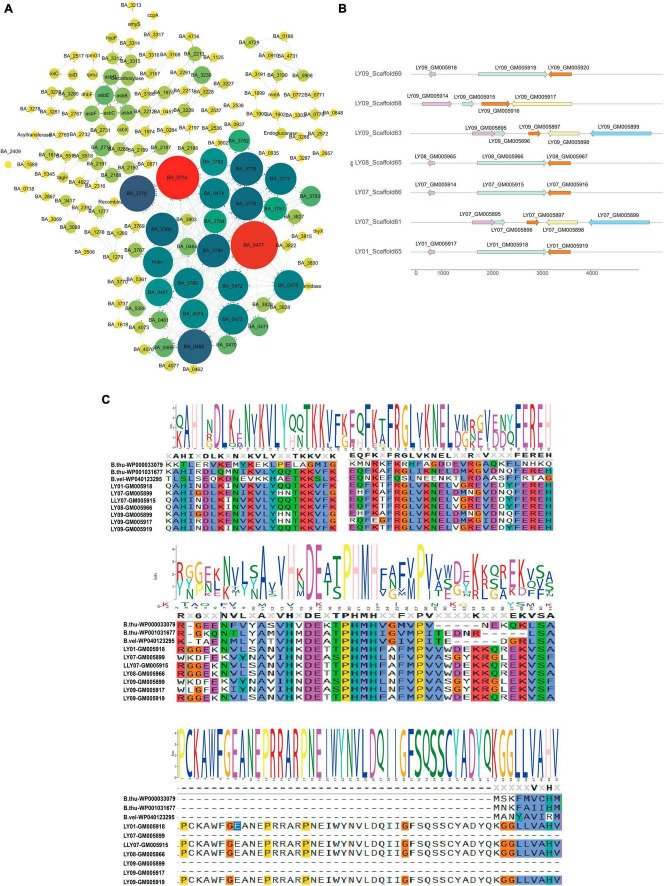
Functions of fast evolving genes and structure of heat-shock protein 20 (*HSP20*) *B. anthracis*, *B. amyloliquefaciens*, *B. thuringiensis, B. velezensis, B. cereus* and *Clostridium botulinum* downloaded from database and the nine *Bacillus* isolates. **(A)** Functions of fast-evolving genes in the 15 strains; **(B)** *HSP20* gene structure of *LY01-LY09*; **(C)** Conserved motifs and loci of *HSP20.*

To understand genetic alterations of the *LY01-LY09* strains and evolutionary forces driving these changes, we examined the interactions among the gene families that have expanded, contracted, and rapidly evolved (Supplementary Figure 6), and identified key genes in gene co-expression networks. The findings indicate that *BA_0477, BA_3774*, and *BA_3775*, which were closely associated with prophage (Supplementary Table 5), were the key genes within the contracted gene families of the *LY01-LY09* strains (Supplementary Figure 7A). The genes belonging to the contracted gene families were primarily associated with phage terminase, phage portal protein, DNA binding, and extracellular region (Supplementary Figure 7B). Similarly, GO enrichment analysis were performed on the expanded gene families, which showed close relationships with DNA recombination, resolvase, N-terminal domain, Tn3 transposase DDE domain, recombinase conserved site and other related factors (Supplementary Figure 7C). To better understand species evolution, we investigated the complete genome sequences of *B. anthracis*, *B. amyloliquefaciens*, *B. thuringiensis, B. velezensis, B. cereus* and *Clostridium botulinum*, together with those of the nine *Bacillus* isolates. By doing so, we intended to explore the interactions among rapidly evolving gene families of the 15 strains. It was discovered that the key genes of these families were the same as those of the contracted gene families in the *LY01-LY09* strains, and all of them were closely associated with prophage (Figure 2A; Supplementary Table 5). The Heat-shock protein 20 (*Hsp20*) family changed significantly in gene number among the rapidly evolving gene families. Structural analysis revealed that the *Hsp20* families of LY09Scaffold63 and LY07LY09Scaffold61 were the most similar in terms of gene number and structure. We thus speculate that they performed similar functions as well (Figure 2B). The protein sequences of the *Hsp20* families in *LY01-LY09*, *B. thuringiensis*, and *B. velezensis* exhibited significant variations due to point mutations and fragment insertions (Figure 2C).

### 3.6 Virulence factors of the *LY01-LY09* strains causing vomiting

**FIGURE 3:**
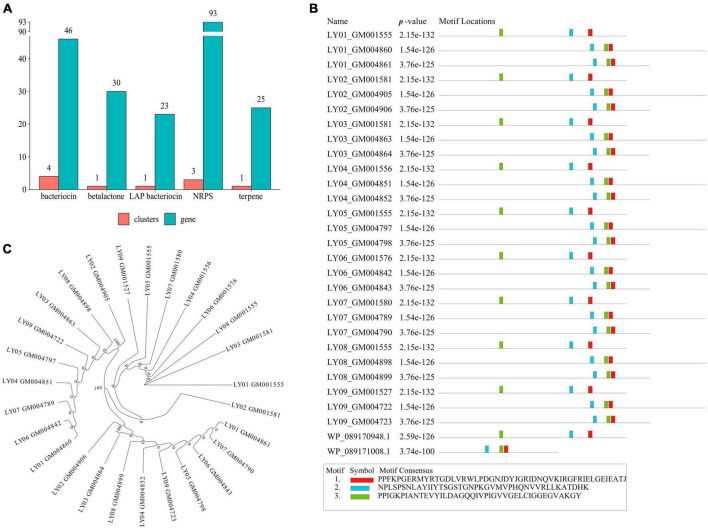
Gene clusters and *NRPS* gene analysis of *B. cereus.* **(A)** Gene cluster analysis of *B. cereus*; **(B)** Motif analysis of the *NRPS* genes; **(C)** Phylogenetic analysis of the *NRPS* genes.

We also observed a similar number of identical gene clusters across the nine *B. cereus* strains. Moreover, these gene clusters mainly focused on *NRPS*, leucine aminopeptidase (*LAP*), bacteriocin, and terpene (Figure 3A), among which the number of the *NRPS* genes was the highest. A further analysis of the *NRPS* genes revealed that Motif-1, Motif-2 and Motif-3 were the shared motifs among the genes (Figure 3B). In addition, the motif sequences were 2-3-1 and 3-2-1, indicating that the *NRPS* gene family in the *LY01-LY09* strains was highly conservative. According to the maximum likelihood-based phylogenetic tree of NRPS proteins, all but one of the 27 NRPS proteins could be assigned to three groups (Figure 3C). Group 1 comprised of eight protein-coding genes, group 2 nine genes, and group 3 also nine genes.

The consistency of virulence factors among strains *LY01-LY09* was further confirmed by analyzing the VFDB database (Table 1). The polysaccharide capsule involved the most virulence genes, totaling 16 in quantity. Capsular polysaccharide is a major virulence determinant for numerous bacteria, as it has the ability to evade host phagocytosis and immune responses, resulting in immunodeficiency and bacterial resistance against host defenses (Wang et al., 2023). Cereulide ranked second in virulence gene number (7 genes). It is an important toxin produced by pathogenic *B. cereus*, and is encoded by the *ces* gene cluster and synthesized by the regulation of RRPS (Kalbhenn et al., 2022). By q-PCR, *hblC, nheB*, and *cesB* were detected but only *cesB* was positive (Supplementary Table 6). In addition, the hemolysin BL gene (*hblA*, *hblC*, *hblD*) and non-hemolytic enterotoxin Nhe gene (*nheC*) associated with diarrhea toxins were discovered in the nine *B. cereus* strains (Table 1). The results showed that *cesB* had a high copy number, but the *hblC* gene was not detected in *B. cereus*.

**TABLE 1:** Analysis of virulence factors.

Classification	Virulence factor	Related gene	Gene number
Enzymes	Sphingomyelinase (SMase)	*sph*	1
Immune escape	*B. cereus* exo-polysaccharide (BPS)	*bpsC*	1
	Polysaccharide capsule	Undetermined	16
Iron to obtain	Bacillibactin	*dhbA, dhbB, dhbC, dhbE, dhbF*	4
	Hal	*hal*	1
	IlsA	*ilsA*	1
Regulation	PagR-XO2	*pagR-XO2*	1
	PlcR-PapR quorum sensing	*papR*	1
		*plcR*	1
	CheA/CheY (Listeria)	*cheA*	1
Toxin	Cereulide	*cesA, cesB, cesC, cesD, cesH, cesP, cesT*	7
	Non-hemolytic enterotoxin (Nhe)	*nheC*	1
Others	O-antigen (Yersinia)	*ddhA*	1

## 4 Discussion

According to annotation analysis of public databases, the metabolic pathways of the nine *B. cereus* isolates were mainly focused on amino acid metabolism, carbohydrate metabolism, membrane transport, caffeine, vitamin metabolism, and energy metabolism. Amino acid metabolism in organisms mainly includes the synthesis of proteins, peptides and other nitrogen-containing substances. Amino acids can also be broken down into α-keto acids, amines and carbon dioxide through deamination, transamination, combined deamination or decarboxylation. The synthesis of *B. cereus* toxins is catalyzed by multiple enzymes. Therefore, the metabolism of amino acids in *B. cereus* has potential functions in regulating the synthesis and expression of virulence factors (Tran et al., 2011; Yu et al., 2011). Carbohydrates function primarily as the energy source for organisms through glycolysis, tricarboxylic acid cycle, pentose phosphate pathway, gluconeogenesis, gluconeogenesis, etc. (Li et al., 2020). The functions of carbohydrates in *LY01-LY09* fell into four categories according to the CAZy database, whose number of genes from high to low were glycosyltransferases (GTs), glycoside hydrolases (GHs), carbohydrate esterolytic enzymes (CEs), and oxidoreductase (AAs). GTs can catalyze the formation of glycosidic bonds between specific sugars and receptors. Their substrates encompass a range of biomolecules, including sugars, proteins, lipids, and other small molecules that are essential for the synthesis of biological compounds (Hsieh et al., 2014). The analysis of the metabolic pathways of *B. cereus* increases our understanding of the mechanism of synthesis and secretion of bacterial toxins.

By comparative genomics, we found more than 99.98% ANI between each pair of the nine *B. cereus* genomes. At the same time, the results of collinearity and SNP analyses also showed high similarity between strains. Therefore, we speculate these nine strains to be the same one, which caused the foodborne outbreaks. The results of phylogenetic and virulence factor analyses were consistent with the above conclusion. Interestingly, phylogenetic analysis revealed that the *LY01-LY09* strains identified in this study was closely related to *B. cereus* and *B. anthracis* (Figure 1). *B. cereus* and *B. anthracis*, which are closely related, are members of the *B. cereus* s.l. group, as evidenced by the high collinearity and similarity in their chromosomes and proteins (Pilo and Frey, 2011). Therefore, it is difficult to discriminate *B. cereus* from *B. anthracis*, particularly for isolates exhibiting unusual biochemical or physiological characteristics. It is usually not enough when relying solely on genome-wide information to differentiate between the two species. Phenotype and plasmid content are also needed to take into consideration (Ehling-Schulz et al., 2019; Lin et al., 2022; Trunet et al., 2023). Phylogenetic analysis revealed that *B. cereus* and *B. thuringiensis* were clustered within the same clade, indicating a close evolutionary relationship. Previous research has indicated that *B. cereus* and *B. thuringiensis* are highly similar in genome. The only difference between the two strains is that *B. thuringiensis* can produce parasporal crystals in the outer spore membrane during spore formation, which is encoded by the insecticidal plasmids. The removal of this plasmid can result in the conversion of *B. thuringiensis* to *B. cereus* (Shu-sen et al., 2007; Venkateswaran et al., 2017). These results further confirm the high genetic similarity and close relationship between diarrheal toxin-producing *B. cereus* and *B. thuringiensis*.

By exploring the alterations in gene families across species, it was found that *B. cereus*, *B. thuringiensis* and *B. anthracis* have expanded significantly. Conversely, the gene families of the isolated *B. cereus* strains have contracted. We speculate that variations in the number of gene families may be responsible for the phenotypic differences of the three *Bacillus* species. This reflects genomic variations among different strains of the same species. To further investigate the relationship among the *LY01-LY09* strains, we constructed an evolutionary tree based on single-copy genes. The analysis revealed that *B. thuringiensis* strains, *B. anthracis* strains, *LY01* and *LY09* formed a closely related clade, which is consistent with conclusions of previous studies. Previous research has shown that *B. cereus* s.l. can be classified into three separate clades. Most *B. cereus* can be classified into two clades: clade A, which is capable of producing emetic toxin, and clade B, which cannot. *B. thuringiensis*, an insect pathogen, mainly belongs to clade B, whereas *B. anthracis* was relatively conservative and restricted to clade A (Ehling-Schulz et al., 2019; Biggel et al., 2022; Carroll et al., 2022). As illustrated in Figure 1, the close clustering pattern between *LY01-LY09* and *B. anthracis* suggests that *LY01-LY09* may be pathogens that are harmful to humans as well. Previous studies have demonstrated that *HSP20* exhibits chaperone activity, which can enhance cellular tolerance to damage and maintain normal cellular metabolism under various stresses, thus improving cell survival rates (Kranzler et al., 2016; Ling et al., 2022; Niu et al., 2022). *LY07* and *LY09* had more than one *Hsp20* genes, some of which undergone point mutations and loss of long amino acids. Therefore, we speculated that the *LY07* and *LY09* strains may exhibit lower susceptibility to environmental stressors such as temperature, demonstrating greater adaptation to adverse conditions in comparison to the *LY01* and *LY08* strains.

Non-ribosomal polypeptide synthetase are the major systemic enzymes that regulate the synthesis of emetic toxins (Dommel et al., 2010). This study identified many *NRPS* genes in the *LY01-LY09* strains, which may suggest that the *LY01-LY09* strains can induce the vomiting-type food poisoning by synthesizing the emetic toxin cereulide regulated by *NRPS*. The *NRPS* biosynthetic machinery is a vast modular multi-enzyme complex that participates in the production of diverse natural products, including toxins, antibiotics, and surfactants. The emetic toxin cereulide is a typical product among these compounds (Bozhüyük et al., 2019). In a previous study, PCR was employed to explore the *NRPS* genes of an emetic strain of *B. cereus*, which confirmed that cereulide was produced via NRPS in *B. cereus* (Toh et al., 2004). *NRPS* plays a key role in the biosynthesis of emetic toxins (cereulide). The gene locus encoding Cereulide synthetase shows the typical structure of the *NRPS* genes clusters, which plays an important role in the synthetic expression of *B. cereus* cereulide (Magarvey et al., 2006; Makarasen et al., 2009). Meanwhile, we have identified seven genes (*cesA, cesB, cesC, cesD, cesH, cesP*, and *cesT*) within the emetic toxin synthetase gene cluster (*ces* gene cluster) in the *LY01-LY09* strains. *CesPTABCD* can form a ces-operon in the megaplasmid pBCE4810, which is transcribed into a 23 kb polycistronic mRNA chain driven by the main promoter p1. Adjacent to *CesPTABCD*, cesH is transcribed as a single cistron by its own promoter PH and encodes a 31 kDa hydrolase (Ehling-Schulz et al., 2015; Lücking et al., 2015). *CesT* is involved in the synthesis of phosphopantetheinyl transferase encoded by *ces*P, which plays a crucial role in initiating non-ribosomal synthesis. In contrast, *cesA* and *cesB* are important in peptide assembly due to their high affinity for Nicotinamide adenine dinucleotide phosphate (NADPH) (Alonzo et al., 2015), while *cesC* and *cesD* are involved in encoding ATP-binding cassette (ABC) transporters (Dommel et al., 2010). The *B. cereus* strains isolated in this study carried a complete *ces* gene-cluster sequence and the diarrheal toxin *nheC*, as well as a substantial number of *NRPS* genes. We speculate that the isolated *B. cereus* regulated the *ces* gene cluster to synthesize emetic toxins mainly via the NRPS system, thereby inducing foodborne illness associated with vomiting (Ehling-Schulz et al., 2006).

## 5 Conclusion

In this study, nine strains of *B. cereus* (*LY01-LY09*) were successfully isolated from food samples of foodborne outbreaks. Their whole genome sequences were obtained by high-throughput sequencing. Based on this, the genetic relationship and gene family of *LY01-LY09* and their related strains were studied via comparative genomics. The results showed that the nine strains belonged to *B. cereus* s.l and they were the same strain. Their genome has unique characteristics compared with *B. thuringiensis* and *B. anthracis*. According to the analysis of virulence factors of *LY01-LY09*, it was found that they all contained emetic toxin and *NRPS* gene clusters. Genomic characteristics of the strains were explained. These results provide insights for further study of the evolutionary relationship of *B. cereus* and the mechanism of toxin synthesis and secretion. This study provides a scientific basis for the prevention and rapid diagnosis of foodborne diseases at the genome level.

## Data availability statement

The original contributions presented in this study are included in this article/Supplementary material, further inquiries can be directed to the corresponding author.

## Author contributions

LZ designed the experiments and applied for fund. SZ, SL, JH, and YW went to the incidence sites to collect food samples and conducted the tests to identify the *B. cereus* strains. YL performed the q-PCR and prepared data. QZ, GL, YC, and JX have worked together to analyze data and wrote the manuscript, in which QZ drafted the Results and Discussion section. GL conducted comparative genome analysis. All authors participated in the rounds of manuscript revision, contributed to the manuscript, and approved its submission and publication.

## Footnotes

1
https://www.genome.jp/kegg

2
http://www.tcdb.org

3
www.phi-base.org

4
http://www.cazy.org/

5
http://www.mgc.ac.cn/VFs/

6
https://meme-suite.org/meme/

## References

[B1] Alonzo D. Magarvey N. Schmeing T. (2015). Characterization of cereulide synthetase, a toxin-producing macromolecular machine. *PLoS One* 10:e0128569. 10.1371/journal.pone.0128569 26042597 PMC4455996

[B2] Anon (1995). *Bacillus cereus in foods. Enumeration and Confirmation. Microbiological Methods.* Rockville: AOAC International.

[B3] Antunes P. Novais C. Peixe L. (2020). Food-to-humans bacterial transmission. *Microbiol Spectr.* 8 161–193. 10.1128/microbiolspec.MTBP-0019-2016 31950894 PMC10810214

[B4] Balloux F. Brønstad Brynildsrud O. van Dorp L. Shaw L. Chen H. Harris K. (2018). From theory to practice: Translating whole-genome sequencing (WGS) into the clinic. *Trends Microbiol*. 26 1035–1048. 10.1016/j.tim.2018.08.004 30193960 PMC6249990

[B5] Biggel M. Jessberger N. Kovac J. Johler S. (2022). Recent paradigm shifts in the perception of the role of *Bacillus thuringiensis* in foodborne disease. *Food Microbiol*. 105:104025. 10.1016/j.fm.2022.104025 35473978

[B6] Bozhüyük K. Micklefield J. Wilkinson B. (2019). Engineering enzymatic assembly lines to produce new antibiotics. *Curr. Opin. Microbiol.* 51 88–96. 10.1016/j.mib.2019.10.007 31743841 PMC6908967

[B7] Carroll L. Cheng R. Wiedmann M. Kovac J. (2022). Keeping up with the *Bacillus cereus* group: Taxonomy through the genomics era and beyond. *Crit Rev Food Sci Nutr*. 62 7677–7702. 10.1080/10408398.2021.1916735 33939559

[B8] Carroll L. Wiedmann M. (2020). Cereulide synthetase acquisition and loss events within the evolutionary history of group III *Bacillus cereus* sensu lato facilitate the transition between emetic and diarrheal foodborne pathogens. *mBio* 11:e01263-20. 10.1128/mBio.01263-20 32843545 PMC7448271

[B9] Castresana J. (2000). Selection of conserved blocks from multiple alignments for their use in phylogenetic analysis. *Mol. Biol. Evol.* 17 540–552. 10.1093/oxfordjournals.molbev.a026334 10742046

[B10] Darriba D. Taboada G. Doallo R. Posada D. (2011). ProtTest 3: Fast selection of best-fit models of protein evolution. *Bioinformatics* 27 1164–1165. 10.1093/bioinformatics/btr088 21335321 PMC5215816

[B11] Dietrich R. Jessberger N. Ehling-Schulz M. Märtlbauer E. Granum P. (2021). The food poisoning toxins of *Bacillus cereus*. *Toxins* 13:98. 10.3390/toxins13020098 33525722 PMC7911051

[B12] Dommel M. Frenzel E. Strasser B. Blöchinger C. Scherer S. Ehling-Schulz M. (2010). Identification of the main promoter directing cereulide biosynthesis in emetic *Bacillus cereus* and its application for real-time monitoring of ces gene expression in foods. *Appl. Environ. Microbiol.* 76 1232–1240. 10.1128/AEM.02317-09 20038713 PMC2820966

[B13] Edgar R. (2004). MUSCLE: Multiple sequence alignment with high accuracy and high throughput. *Nucleic Acids Res*. 32 1792–1797. 10.1093/nar/gkh340 15034147 PMC390337

[B14] Ehling-Schulz M. Frenzel E. Gohar M. (2015). Food-bacteria interplay: Pathometabolism of emetic *Bacillus cereus*. *Front. Microbiol.* 6:704. 10.3389/fmicb.2015.00704 26236290 PMC4500953

[B15] Ehling-Schulz M. Fricker M. Grallert H. Rieck P. Wagner M. Scherer S. (2006). Cereulide synthetase gene cluster from emetic *Bacillus cereus*: Structure and location on a mega virulence plasmid related to *Bacillus anthracis* toxin plasmid pXO1. *BMC Microbiol*. 6:20. 10.1186/1471-2180-6-20 16512902 PMC1459170

[B16] Ehling-Schulz M. Lereclus D. Koehler T. (2019). The *Bacillus cereus* group: *Bacillus species* with pathogenic potential. *Microbiol. Spectr.* 7. 10.1128/microbiolspec.gpp3-0032-2018 PMC6530592 31111815

[B17] Emms D. Kelly S. (2019). OrthoFinder: Phylogenetic orthology inference for comparative genomics. *Genome Biol*. 20:238. 10.1186/s13059-019-1832-y 31727128 PMC6857279

[B18] He S. Shi X. (2021). Microbial food safety in China: Past, present, and future. *Foodborne Pathog. Dis.* 18, 510–518. 10.1089/fpd.2021.0009 34242111

[B19] Hsieh Y. Chiu H. Huang Y. Fun H. Lu C. Li Y. (2014). Purification, crystallization and preliminary X-ray crystallographic analysis of glycosyltransferase-1 from *Bacillus cereus*. *Acta Crystallogr. F Struct. Biol. Commun*. 70(Pt 9), 1228–1231. 10.1107/S2053230X14014629 25195897 PMC4157424

[B20] Jain C. Rodriguez-R L. Phillippy A. Konstantinidis K. Aluru S. (2018). High throughput ANI analysis of 90K prokaryotic genomes reveals clear species boundaries. *Nat. Commun.* 9:5114. 10.1038/s41467-018-07641-9 30504855 PMC6269478

[B21] Josephs-Spaulding J. Beeler E. Singh O. (2016). Human microbiome versus food-borne pathogens: Friend or foe. *Appl. Microbiol. Biotechnol.* 100 4845–4863. 10.1007/s00253-016-7523-7 27102132

[B22] Kalbhenn E. Kranzler M. Gacek-Matthews A. Grass G. Stark T. Frenzel E. (2022). Impact of a novel pagr-like transcriptional regulator on cereulide toxin synthesis in emetic *Bacillus cereus*. *Int. J. Mol. Sci*. 23:11479. 10.3390/ijms231911479 36232797 PMC9570423

[B23] Kotiranta A. Lounatmaa K. Haapasalo M. (2000). Epidemiology and pathogenesis of *Bacillus cereus* infections. *Microbes Infect*. 2 189–198. 10.1016/s1286-4579(00)00269-0 10742691

[B24] Kranzler M. Stollewerk K. Rouzeau-Szynalski K. Blayo L. Sulyok M. Ehling-Schulz M. (2016). Temperature exerts control of *Bacillus cereus* emetic toxin production on post-transcriptional levels. *Front. Microbiol.* 7:1640. 10.3389/fmicb.2016.01640 27826288 PMC5078297

[B25] Krzywinski M. Schein J. Birol I. Connors J. Gascoyne R. Horsman D. (2009). Circos: An information aesthetic for comparative genomics. *Genome Res*. 19 1639–1645. 10.1101/gr.092759.109 19541911 PMC2752132

[B26] Kurtz S. Phillippy A. Delcher A. Smoot M. Shumway M. Antonescu C. (2004). Versatile and open software for comparing large genomes. *Genome Biol*. 5:R12. 10.1186/gb-2004-5-2-r12 14759262 PMC395750

[B27] Li W. Cui Q. Bai L. Fu P. Han H. Liu J. (2021). Application of whole-genome sequencing in the national molecular tracing network for foodborne disease surveillance in China. *Foodborne Pathog. Dis.* 18 538–546. 10.1089/fpd.2020.2908 34339263

[B28] Li Y. Zhao M. Chen W. Du H. Xie X. Wang D. (2020). Comparative transcriptomic analysis reveals that multiple hormone signal transduction and carbohydrate metabolic pathways are affected by *Bacillus cereus* in *Nicotiana tabacum*. *Genomics* 112 4254–4267. 10.1016/j.ygeno.2020.07.022 32679071

[B29] Lin S. Liao Y. C. (2013). CISA: Contig integrator for sequence assembly of bacterial genomes. *PLoS One* 8:e60843. 10.1371/journal.pone.0060843 23556006 PMC3610655

[B30] Lin Y. Briandet R. Kovács Á (2022). *Bacillus cereus* sensu lato biofilm formation and its ecological importance. *Biofilm* 4:100070. 10.1016/j.bioflm.2022.100070 35243332 PMC8861577

[B31] Ling Y. Ling Z. Zhao R. (2022). Construction of a heat-resistant strain of Lentinus edodes by fungal Hsp20 protein overexpression and genetic transformation. *Front. Microbiol.* 13:1009885. 10.3389/fmicb.2022.1009885 36478857 PMC9721462

[B32] Liu B. Zheng D. Zhou S. Chen L. Yang J. (2022). VFDB 2022: A general classification scheme for bacterial virulence factors. *Nucleic Acids Res.* 50 D912–D917. 10.1093/nar/gkab1107 34850947 PMC8728188

[B33] Lücking G. Frenzel E. Rütschle A. Marxen S. Stark T. Hofmann T. (2015). Ces locus embedded proteins control the non-ribosomal synthesis of the cereulide toxin in emetic *Bacillus cereus* on multiple levels. *Front. Microbiol.* 6:1101. 10.3389/fmicb.2015.01101 26528255 PMC4602138

[B34] Magarvey N. Ehling-Schulz M. Walsh C. (2006). Characterization of the cereulide NRPS alpha-hydroxy acid specifying modules: Activation of alpha-keto acids and chiral reduction on the assembly line. *J. Am. Chem. Soc.* 128 10698–10699. 10.1021/ja0640187 16910662

[B35] Makarasen A. Yoza K. Isobe M. (2009). Higher structure of cereulide, an emetic toxin from *Bacillus cereus*, and special comparison with valinomycin, an antibiotic from *Streptomyces fulvissimus*. *Chem. Asian J*. 4 688–698. 10.1002/asia.200900011 19347893

[B36] Marxen S. Stark T. Rütschle A. Lücking G. Frenzel E. Scherer S. (2015). Depsipeptide intermediates interrogate proposed biosynthesis of cereulide, the emetic toxin of *Bacillus cereus*. *Sci. Rep.* 5:10637. 10.1038/srep10637 26013201 PMC4445039

[B37] Niu H. Yang M. Qi Y. Liu Y. Wang X. Dong Q. (2022). Heat shock in *Cronobacter sakazakii* induces direct protection and cross-protection against simulated gastric fluid stress. *Food Microbiol*. 103:103948. 10.1016/j.fm.2021.103948 35082065

[B38] Pilo P. Frey J. (2011). *Bacillus anthracis*: Molecular taxonomy, population genetics, phylogeny and patho-evolution. *Infect. Genet. Evol.* 11 1218–1224. 10.1016/j.meegid.2011.05.013 21640849

[B39] Pires S. Desta B. Mughini-Gras L. Mmbaga B. Fayemi O. Salvador E. (2021). Burden of foodborne diseases: Think global, act local. *Curr. Opin. Food Sci.* 39 152–159. 10.1016/j.cofs.2021.01.006 34178607 PMC8216060

[B40] Premkrishnan B. Heinle C. Uchida A. Purbojati R. Kushwaha K. Putra A. (2021). The genomic characterisation and comparison of *Bacillus cereus* strains isolated from indoor air. *Gut Pathog*. 13:6. 10.1186/s13099-021-00399-4 33516253 PMC7847026

[B41] Sánchez Chica J. Correa M. Aceves-Diez A. Rasschaert G. Heyndrickx M. Castañeda-Sandoval L. (2020). Genomic and toxigenic heterogeneity of bacillus cereus sensu lato isolated from ready-to-eat foods and powdered milk in day care centers in Colombia. *Foodborne Pathog. Dis*. 17 340–347. 10.1089/fpd.2019.2709 31738585

[B42] Shu-sen S. Li-li J. Fang W. (2007). Study on genetic relationship between *Bacillus thuringiensis* and *Bacillus cereus*. *Chin. J. Public Health* 23 321–323.

[B43] Stamatakis A. (2014). RAxML version 8: A tool for phylogenetic analysis and post-analysis of large phylogenies. *Bioinformatics* 30 1312–1313. 10.1093/bioinformatics/btu033 24451623 PMC3998144

[B44] Stenfors Arnesen L. Fagerlund A. Granum P. (2008). From soil to gut: *Bacillus cereus* and its food poisoning toxins. *FEMS Microbiol. Rev*. 32 579–606. 10.1111/j.1574-6976.2008.00112.x 18422617

[B45] Toh M. Moffitt M. Henrichsen L. Raftery M. Barrow K. Cox J. (2004). Cereulide, the emetic toxin of *Bacillus cereus*, is putatively a product of nonribosomal peptide synthesis. *J Appl Microbiol.* 97 992–1000. 10.1111/j.1365-2672.2004.02381.x 15479414

[B46] Tran S. Guillemet E. Ngo-Camus M. Clybouw C. Puhar A. Moris A. (2011). Haemolysin II is a *Bacillus cereus* virulence factor that induces apoptosis of macrophages. *Cell Microbiol.* 13 92–108. 10.1111/j.1462-5822.2010.01522.x 20731668

[B47] Trunet C. Cauquil A. Hymery N. Koullen L. Postollec F. Coroller L. (2023). Are *Bacillus thuringiensis* strains like any other *Bacillus cereus* strains? Phenotypic-based tools to locate *Bacillus thuringiensis* in the diversity of the *Bacillus cereus* sensu lato group. *Res. Microbiol.* 174:104077. 10.1016/j.resmic.2023.104077 37149077

[B48] Venkateswaran K. Singh N. Checinska Sielaff A. Pope R. Bergman N. van Tongeren S. (2017). Non-toxin-producing *Bacillus cereus* strains belonging to the B. Anthracis clade isolated from the International Space Station. *mSystems* 2:e00021-17. 10.1128/mSystems.00021-17 28680972 PMC5487513

[B49] Wang Y. J. Li L. Yu J. Hu H. Y. Liu Z. X. Jiang W. J. (2023). Imaging of *Escherichia coli* K5 and glycosaminoglycan precursors via targeted metabolic labeling of capsular polysaccharides in bacteria. *Sci. Adv.* 9:eade4770. 10.1126/sciadv.ade4770 36800421 PMC9937569

[B50] Yu C. Wang Y. Xu C. He J. Zhang Q. Yu Z. (2011). [Analyze and compare metabolic pathways of *Bacillus cereus* group]. *Yi Chuan* 33 1057–1066. 10.3724/sp.j.1005.2011.01057 21993280

[B51] Zhou G. Liu H. He J. Yuan Y. Yuan Z. (2008). The occurrence of *Bacillus cereus*, *B. thuringiensis* and *B. mycoides* in Chinese pasteurized full fat milk. *Int J Food Microbiol.* 121 195–200. 10.1016/j.ijfoodmicro.2007.11.028 18077041

