# Supplementary material for: Genomic characterization of Bacillus cereus isolated from food poisoning cases revealed the mechanism of toxin production
Source: Front Microbiol. 2024 Jan 12;14:1238799. doi: 10.3389/fmicb.2023.1238799 (PMC10822677; doi:10.3389/fmicb.2023.1238799)
Supplement: Supplementary file 1 [file Data_Sheet_1.pdf]

Supplementary Material

Figure Legends:

Supplementary Figure 1. Colony morphology and microscopic observation of *Bacillus cereus* (*B. cereus*). A. Colony morphology of *B. cereus* on MYP mediums (red) and nutrient agar (white); B. Microstructure of *B. cereus* after Gram staining.

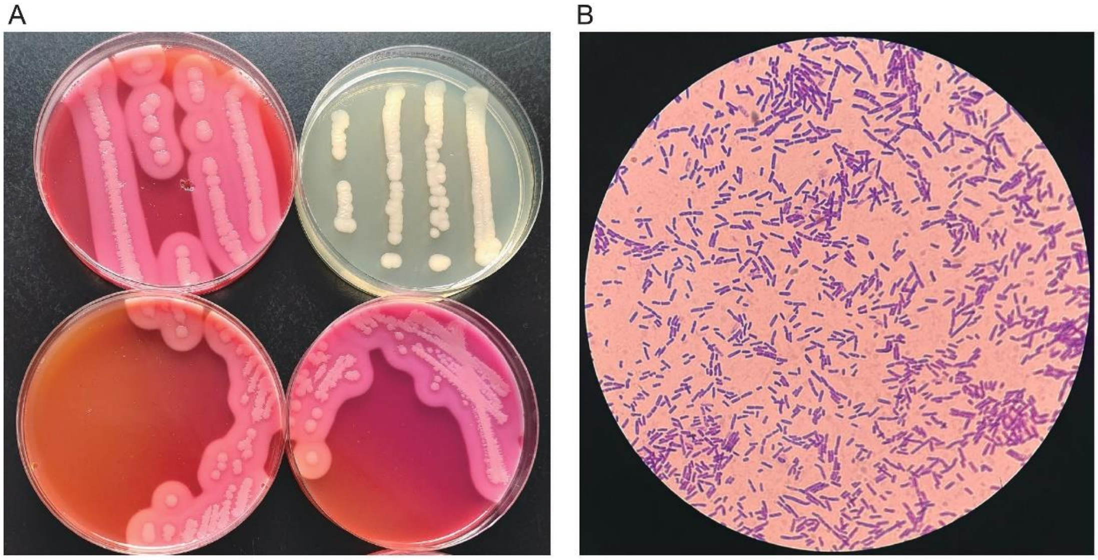

Supplementary Figure 2: Genome structural annotation of the LY01-LY09 strains. A: Repeat sequences in the genome. B: Non-coding RNA in the genome. C: Number of CRISPR, genomic island (Gis), and prophage in the genome.

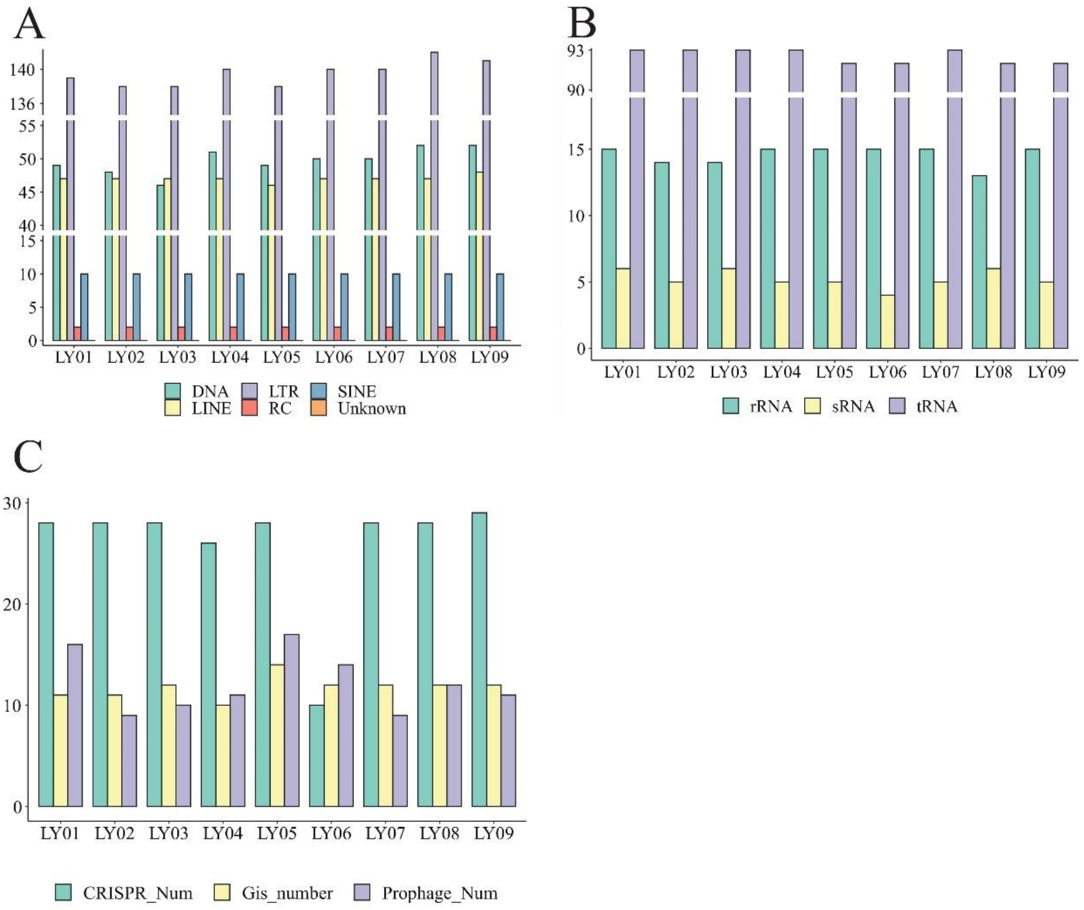

Supplementary Figure 3: Annotations of the LY01-LY09 genomes based on the following databases: KEGG (A), TCDB (B), CAZy (C) and PHI (D).

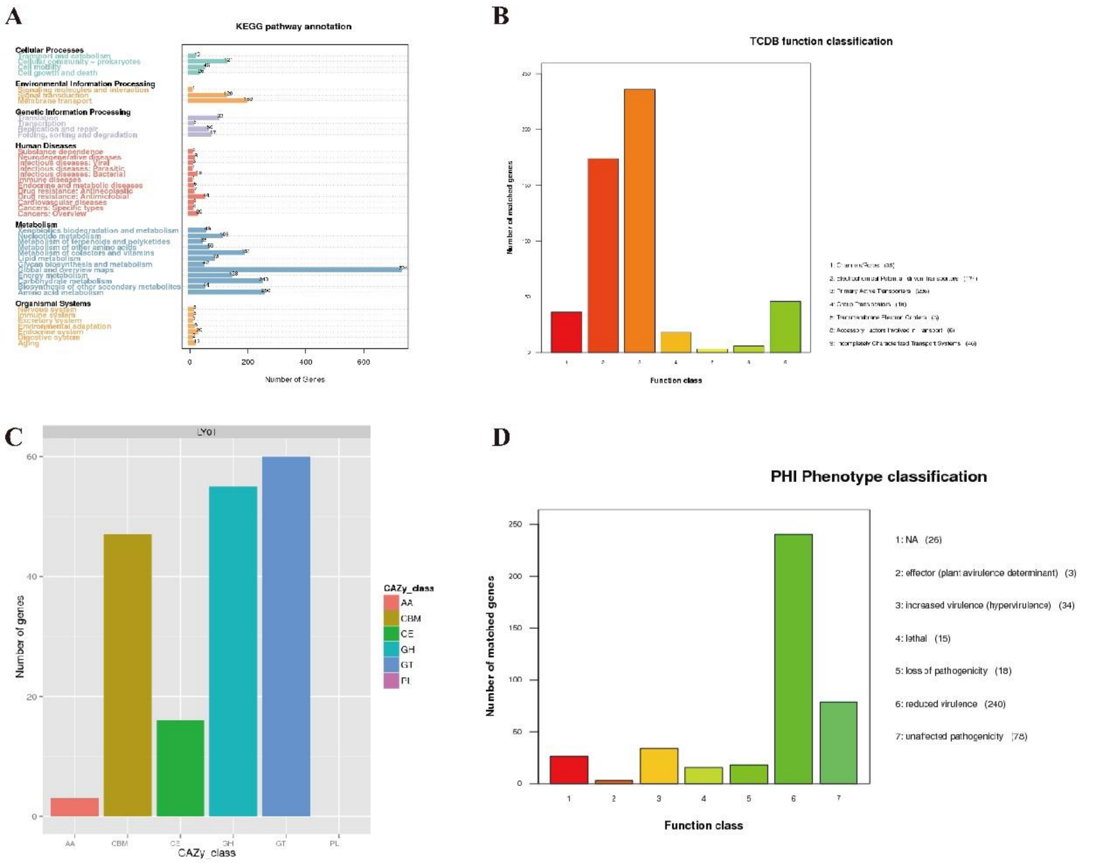

Supplementary Figure 4: Genome collinearity analysis between LY02 and a reference *B. cereus* strain, as well LY02 and LY01.

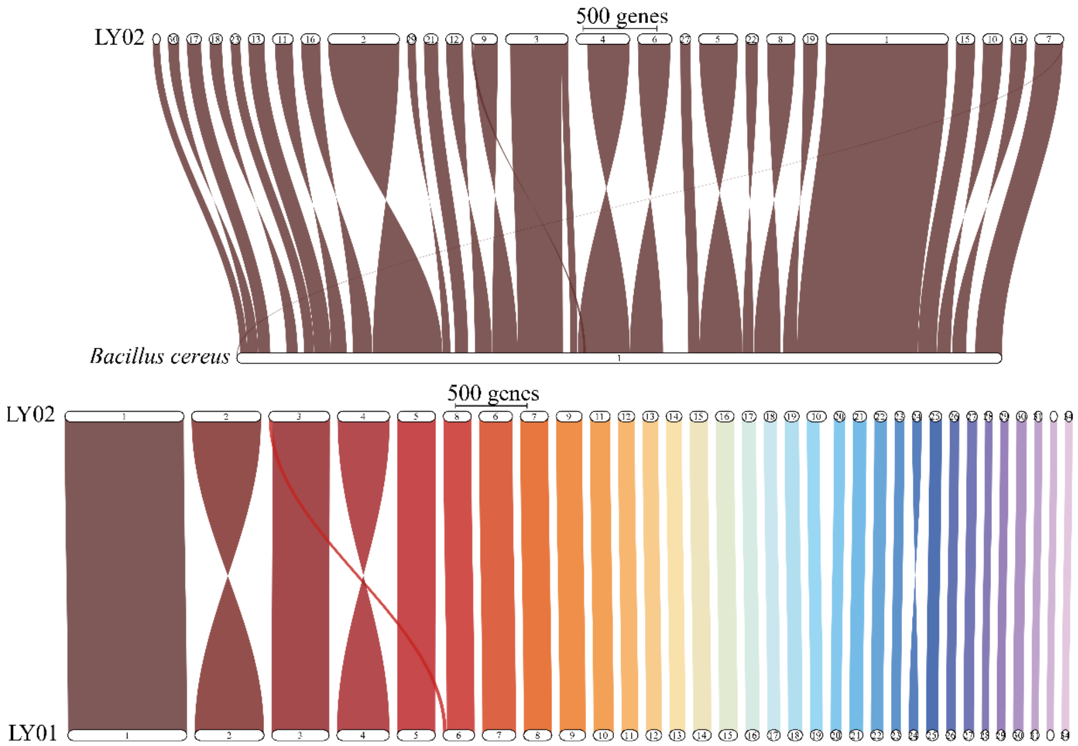

Supplementary Figure 5 : Comparisons of genomic sequences between *LY03* and eight other strains. For the circles, from the outermost to the innermost are: genome karyotype of LY03, and the distribution of SNPs (single nucleotide polymorphisms) and Indels (insertions and deletions) on the genomes of *LY01*, *LY02*, *LY04*, *LY05*, *LY06*, *LY07*, *LY08* and *LY09* strains.

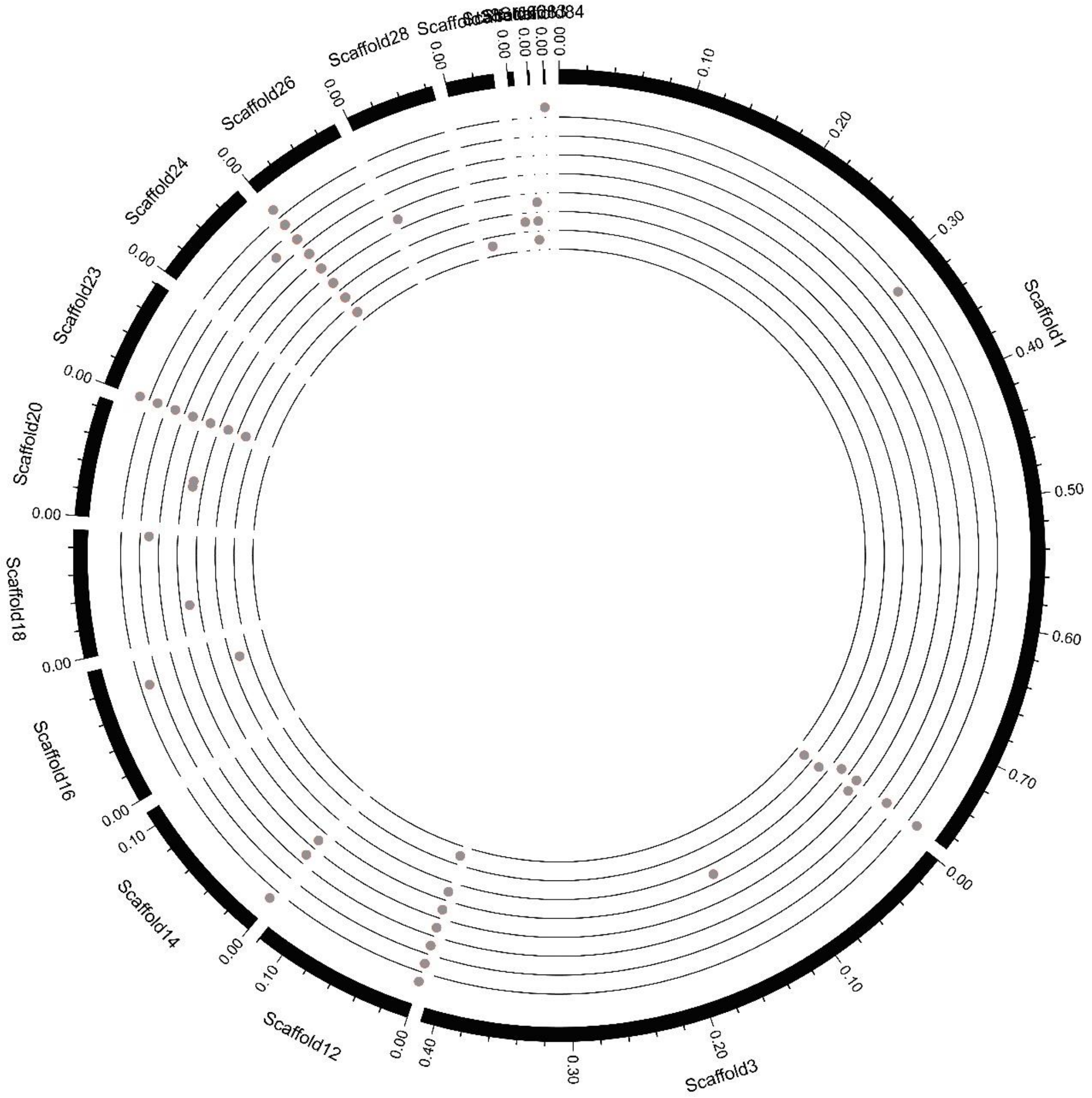

Supplementary Figure 6: Contraction and Expansion analysis of the gene families in *B. anthracis*, *B. amyloliquefaciens*, *B. thuringiensis, B. velezensis, B. cereus* and *Clostridium botulinum* downloaded from database and the nine *Bacillus* isolates.

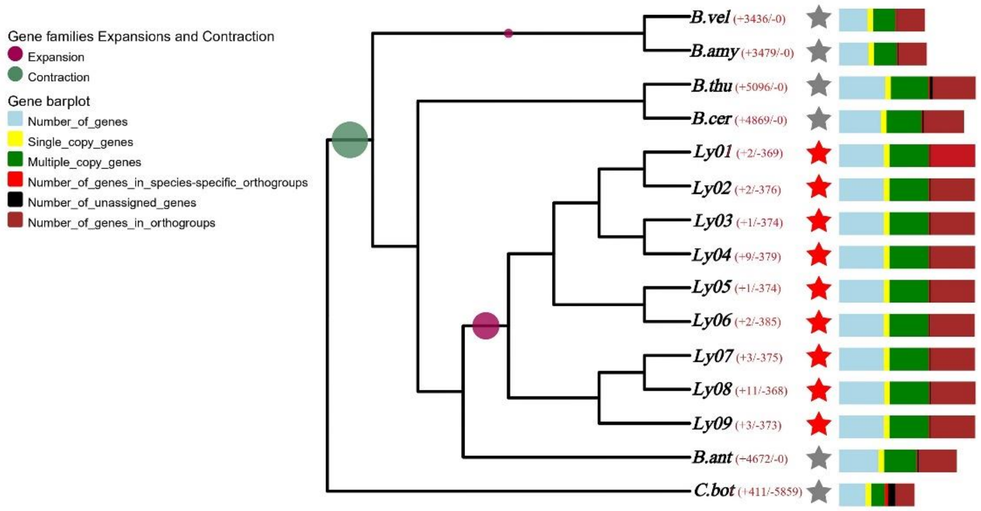

Supplementary Figure 7: Functional analysis of contracted and expanded gene families in the LY01-LY09 strains A: Regulatory network of contracted gene families in the LY01-LY09 strains. B: GO enrichment of contracted families in the LY01-LY09 strains. C: GO enrichment analysis of expanded families in the LY01-LY09 strains.

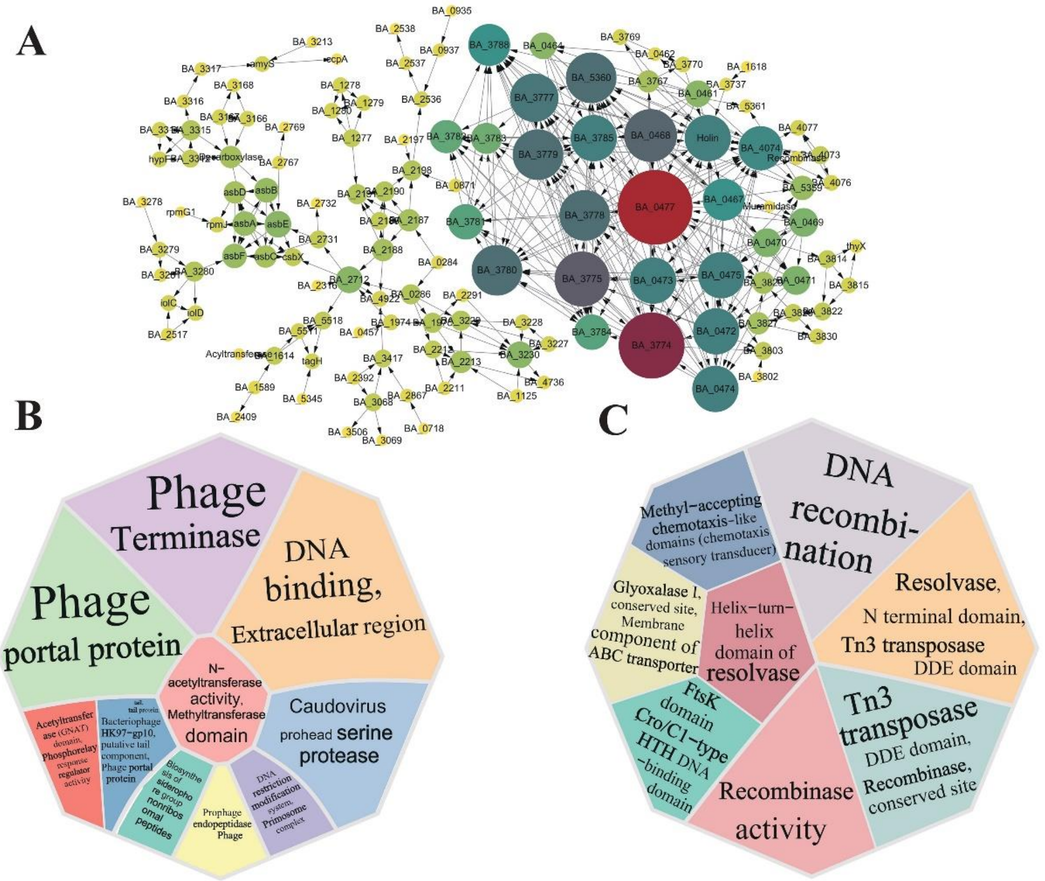

Supplementary Table 1 Symptoms and colony forming unit (CFU) counts of food samples

Outbreak	Case (number)	Nausea	Vomiting	CFU Count (/g)
Ziyun County	10	2	9	rice noodle 6.2×10^8
Ziyun County	5	5	5	rice noodle 1.1×10^8
Ziyun County	3	3	3	rice noodle 3.0×10^5
Tongren City	5	1	1	steamed bread 6.3×10^4
				stir fried pork with potato 5.6×10^5
				maize congee 8.1×10^6
				steamed rice 5.3×10^5
				rice noodle 1.9×10^5

Supplementary Table 2 Physiological and biochemical experiments on *B. cereus*

Feature	character
Gram reaction	+
Lysozyme-resistant	+
Acid produced from mannitol	-
Anaerobic utilization of glucose	+
Starch hydrolysis	+
Reduction of nitrate	+
V-P reaction	+
Citrate utilization	+
Rhizoid growth	-
Motility	+

+, positive; -, negative

Supplementary Table 3 Genome overview

Sample Name	Total number (>500bp)	Overall length (bp)	N50(bp)	GC%	Gene length (bp)	Average gene length (bp)	Proportion of genes (%)	Number of contigs
LY01	91	5,645,608	169,152	35.25	4,764,972	799	84.4	91
LY02	89	5,643,860	169,166	35.25	4,764,186	799	84.41	89
LY03	89	5,643,052	169,172	35.25	4,762,311	799	84.39	89
LY04	89	5,649,453	169,172	35.25	4,766,961	799	84.38	89
LY05	92	5,641,597	169,172	35.25	4,761,279	799	84.4	92
LY06	92	5,642,796	169,164	35.25	4,758,888	800	84.34	92
LY07	90	5,643,289	169,172	35.25	4,762,332	799	84.39	90
LY08	92	5,680,067	169,172	35.23	4,792,617	798	84.38	92
LY09	94	5,644,293	169,172	35.25	4,763,493	799	84.39	94

Supplementary Table 4 Average nucleotide identity (ANI) values between genomes of the *B. cereus* strains

Query	Reference	ANI
LY01	LY02	99.9884
LY01	LY03	99.9968
LY01	LY04	99.9995
LY01	LY05	99.9998
LY01	LY06	99.9993
LY01	LY07	99.9996
LY01	LY08	99.9995
LY01	LY09	99.9994
LY02	LY03	99.9958
LY02	LY04	99.9879
LY02	LY05	99.988
LY02	LY06	99.9877
LY02	LY07	99.988
LY02	LY08	99.9864
LY02	LY09	99.9978
LY03	LY04	99.999
LY03	LY05	99.9992
LY03	LY06	99.9987
LY03	LY07	99.9991
LY03	LY08	99.9986
LY03	LY09	99.9985
LY04	LY05	99.9995
LY04	LY06	99.999
LY04	LY07	99.9994
LY04	LY08	99.9993
LY04	LY09	99.9981
LY05	LY06	99.9994
LY05	LY07	99.9996
LY05	LY08	99.9995
LY05	LY09	99.9995
LY06	LY07	99.9983
LY06	LY08	99.9993
LY06	LY09	99.9971
LY07	LY08	99.9992
LY07	LY09	99.9994
LY08	LY09	99.9967

Supplementary Table 5 Key gene information

gene id	OG_id	gene symbol	Uniprot_id	Proteins	Descriptions
bacillus_anthracis_757	OG0005708	BA_0477	A0A347ZYU4	Prophage	Putative prophage LambdaBa04, tape measure protein
Bacillus_cereus_4132	OG0005708	BA_3774	A0A6H3ALE6	Phage Tail Tape Measure protein	Putative prophage LambdaBa01, membrane protein
Bacillus_thuringiensis_4060	OG0005708	BA_3775	A0A6L7H158	Prophage	Conserved domain protein
bacillus_anthracis_3972	OG0007222	BA_0468	A0A6H3AAE3	Phage Capsid protein	Putative prophage LambdaBa04, major capsid protein

Supplementary Table 6 Cycle threshold (Ct) for q-PCR detected virulence factors

Sample Name	*cesB*	*hblC*
LY01	18.207	-
LY02	18.884	-
LY03	15.817	-
LY04	14.901	-
LY05	15.284	-
LY06	15.804	-
LY07	16.437	-
LY08	14.177	-
LY09	16.255	-
Positive Control	27.554	30.002
